# Identification of comutation in signaling pathways to predict the clinical outcomes of immunotherapy

**DOI:** 10.1186/s12967-022-03836-3

**Published:** 2022-12-23

**Authors:** Jiayue Qiu, Xiangmei Li, Yalan He, Qian Wang, Ji Li, Jiashuo Wu, Ying Jiang, Junwei Han

**Affiliations:** 1 College of Bioinformatics Science and Technology, Harbin Medical University, 157 BaoJian Road, Harbin, 150081 People’s Republic of China; 2 College of Basic Medical Science, Heilongjiang University of Chinese Medicine, Harbin, 150040 People’s Republic of China

**Keywords:** Pathway analysis, Comutation, Tumour mutational burden, Immunotherapy biomarker

## Abstract

**Background:**

Immune checkpoint blockades (ICBs) have emerged as a promising treatment for cancer. Recently, tumour mutational burden (TMB) and neoantigen load (NAL) have been proposed to be potential biomarkers to predict the efficacy of ICB; however, they were limited by difficulties in defining the cut-off values and inconsistent detection platforms. Therefore, it is critical to identify more effective predictive biomarkers for screening patients who will potentially benefit from immunotherapy. In this study, we aimed to identify comutated signaling pathways to predict the clinical outcomes of immunotherapy.

**Methods:**

Here, we comprehensively analysed the signaling pathway mutation status of 9763 samples across 33 different cancer types from The Cancer Genome Atlas (TCGA) by mapping the somatic mutations to the pathways. We then explored the comutated pathways that were associated with increased TMB and NAL by using receiver operating characteristic (ROC) curve analysis and multiple linear regressions.

**Results:**

Our results revealed that comutation of the Spliceosome (Sp) pathway and Hedgehog (He) signaling pathway (defined as SpHe-comut^+^) could be used as a predictor of increased TMB and NAL and was associated with increased levels of immune-related signatures. In seven independent immunotherapy cohorts, we validated that SpHe-comut^+^ patients exhibited a longer overall survival (OS) or progression-free survival (PFS) and a higher objective response rate (ORR) than SpHe-comut^−^ patients. Moreover, a combination of SpHe-comut status with PD-L1 expression further improved the predictive value for ICB therapy.

**Conclusion:**

Overall, SpHe-comut^+^ was demonstrated to be an effective predictor of immunotherapeutic benefit in seven independent immunotherapy cohorts and may serve as a potential and convenient biomarker for the clinical application of ICB therapy.

**Supplementary Information:**

The online version contains supplementary material available at 10.1186/s12967-022-03836-3.

## Introduction

In recent years, immunotherapy with immune checkpoint blockades (ICBs) has emerged as a promising treatment for advanced-stage cancer. There are three main types of ICB therapies, including inhibition of programmed cell death 1 (PD-1) or ligand 1 (PD-L1) and cytotoxic T-lymphocyte antigen-4 (CTLA-4), which have been approved by the FDA for a range of solid tumours [1, 2]. However, only a small subset of patients can benefit from ICBs, and the clinical application of ICBs is still limited. Thus, it is necessary to identify new biomarkers to screen patients who may respond to ICB therapy.

Tumour mutational burden (TMB), defined as the total number of somatic mutations per coding area of the genome in a tumour [3], has gained increasing recognition as a clinically predictive biomarker for immunotherapy response in several cancer types, including melanoma, non-small cell lung cancer (NSCLC), and urothelial carcinoma [4, 5]. However, TMB varies across different cancer types because of the heterogeneity of cancer. Moreover, increasing evidence indicates that TMB assessment and bioinformatics interpretation are also different across various targeted sequencing panels [6]. Therefore, an optimal threshold for classifying a cancer patient into TMB-high or TMB-low in ICB treatment remains unclear [7], which limits TMB as a biomarker for clinical application. Moreover, neoantigen load (NAL) is another potential ICB biomarker: Anagnostou et al. reported that ICB had significant therapeutic effects against tumours with increased mutation-associated neoantigen load [8]. Recent studies have shown that somatic mutations can give rise to neoepitopes, which may serve as neoantigens allowing for enhanced immunogenicity [9, 10]. However, NAL has the same limitation as TMB, which also restricts it as an effective biomarker for ICB [11].

Previous studies have demonstrated that mutations in specific genes may be associated with ICB efficacy [12, 13]. For example, Boichard et al. found that APOBEC-related mutagenesis increases immunogenicity, which was correlated with responses to immunotherapy [14]. Alterations in DNA damage response (DDR) genes are associated with increased TMB and tumour infiltration by T cells [15]. Pan et al. revealed that a 52-gene mutation signature could predict immunotherapy benefits in NSCLC [13]. These studies are mainly based on the gene level; nevertheless, exploring the impacts of mutations on immunotherapy at the pathway level will provide us with more robust results than at the gene level because of tumour heterogeneity. This may be because even if patients have different genetic mutations, these mutated genes tend to be ultimately involved in certain pathways [16, 17].

Thus, several pathway-based biomarker identification methods have been developed. Li et al. revealed that substantial alterations of genes in the Notch pathway may predict the prognosis of NSCLC patients [18]. Another study found that mutations in the PI3K-AKT-mTOR pathway can serve as predictors of the efficacy of immunotherapy in gastric adenocarcinoma [19]. Wang et al. suggested that the co-occurrence of alterations in the related pathways may help elucidate functionally relevant mechanisms that might inform treatment options, and they thus identified that comutation in some DDR pathways can serve as a potential biomarker for ICB [20]. However, they only focused on DDR-related pathways and did not consider other essential pathways, such as signaling pathways. Signaling pathways control essential biological progression, including molecular mechanisms, disease states, and drug responses [21–26]. Mutations in specific signaling pathways, for example, the NOTCH signaling pathway, have been proposed to be associated with the response to ICB in many cancer patients. Nevertheless, comutations in the signaling pathways of immunotherapy remain to be elucidated.

In this study, we comprehensively analysed the signaling pathway mutation status of 9763 samples across 33 different cancer types from The Cancer Genome Atlas (TCGA) to explore the association between the comutated pathway and immunotherapy response. ROC curve and multiple linear regression analyses were performed to determine the optimal combinations of comutated signaling pathways associated with increased TMB and NAL. Moreover, we further explored the associations between comutation in signaling pathways and the efficacy of immunotherapy. Collectively, we aimed to identify comutated signaling pathways to predict the clinical outcomes of immunotherapy, which may provide a potential and convenient biomarker for the future clinical application of immunotherapy.

## Materials and methods

### Data collection

We first collected 9763 cancer patients across 33 cancer types from the GDC TCGA data portal (https://portal.gdc.cancer.gov/), and their somatic mutations (whole-exome sequencing), gene expression (RNA-seq), and clinical data (sex, age, survival information, etc.) were downloaded accordingly. Then, the corresponding neoantigen data of 5446 TCGA cancer patients across 20 cancer types were downloaded from the TCIA (https://tcia.at/neoantigens) [27], and the microsatellite instability (MSI) data from 5930 TCGA cancer patients across 18 cancer types were obtained from the research of Hause et al. [28]. To further validate the association between comutated signaling pathways and the clinical benefit of ICBs, we retrieved seven clinical cohorts treated with ICBs from the published studies: (1) the Inova cohort includes 50 advanced melanoma patients treated with anti-PD-(L)1 or plus anti-CTLA-4 therapy [29]; (2) the Rizvi cohort includes 34 patients with NSCLC treated with anti-PD-1 therapy, with the tumour tissues profiled with the MSKCC panel [30]; (3) the Allen and Snyder cohorts respectively include 110 and 64 patients with melanoma who received anti-CTLA-4 therapy [31, 32]; (4) the Hellmann cohort consists of 75 patients with NSCLC treated with anti-PD-1 plus anti-CTLA-4 therapy [33]; (5) the Miao cohort contains 284 patients with mixed solid tumours (including non-small cell lung cancer, melanoma, head and neck cancer, bladder cancer, and clear cell renal cell carcinoma) treated with anti-CTLA-4 or PD-1/PD-L1 inhibitors [34]; (6) the MSKCC cohort, which was part of a pan-cancer study for targeted sequencing, including 1,661 characterized patients treated with anti-CTLA-4 or PD-1/PD-L1 inhibitors [35]. Patients’ responses to immunotherapy were based on definitions consistent with how they were evaluated in the above cohorts. For the Inova, Allen, Snyder, and Miao cohorts, the tumour immunotherapy response was defined by the Response Evaluation Criteria in Solid Tumours 1.1 (RECIST 1.1), and patients who experienced a complete response (CR) or partial response (PR) were classified as responders; patients who experienced stable disease (SD) or progressive disease (PD) were classified as non-responders. For the Rizvi and Hellmann cohorts, patients were classified as having a durable clinical benefit (DCB) (complete response [CR]/partial response [PR] or stable disease [SD] that lasted > 6 months) or no durable benefit (NDB) (progression of the disease [PD] or SD that lasted ≤ 6 months). Detailed information regarding the above cohorts is shown in Additional file 1: Table S1. We also summarized the details for the time period and dose selection of the above cohorts in Additional file 2: Table S2.

We then downloaded the Kyoto Encyclopedia of Genes and Genomes (KEGG) pathways from the MsigDB database [36] and manually extracted 68 canonical signaling pathways.

### Identifying the comutated pathways associated with high TMB and high NAL

Using TCGA somatic mutation data, we constructed a non-silent gene binary mutation matrix, in which each column represents a sample and each row represents a mutated gene. If a gene mutation occurred in one sample, the element was 1; otherwise, it was 0. In our study, we extracted only non-silent somatic mutations in the genomic coding regions, including missense mutations, nonsense mutations, insertions, deletions, and splice mutations. Genes with mutation frequencies greater than 1% were considered. Then, we mapped the mutated genes into signaling pathways, and if a pathway involved at least one mutated gene, the pathway was defined as mutated. Pathways with a mutation frequency of at least 1% were retained. For each pathway, we used the multivariate Cox proportional hazard model to estimate the association between the pathway mutation status and overall survival (OS) by accounting for known covariates such as patient age, sex, and cancer type. The statistical significance levels (p values) of pathways were then corrected with the false discovery rate (FDR) method proposed by Benjamini and Hochberg [37]. Then, the pathways with FDR < 0.05 were retained for further analysis.

High TMB and NAL were proposed to be associated with an improved response to ICB treatment [38]. In this study, TMB was calculated as the total number of non-silent somatic mutations per coding area of the genome in a tumour, and the NAL data were downloaded from the TCIA (https://tcia.at/neoantigens). We intended to identify the comutated pathways associated with high TMB and NAL. To do this, we first divided the TCGA cancer patients into high TMB/NAL and low TMB/NAL groups according to the top quartile of TMB/NAL levels. Receiver operating characteristic (ROC) curve analysis was then used to determine the cut-off points for pathway mutation counts based on the highest Youden index. According to the cut-off of pathway mutation counts, we further screened which pathways were comutated. Multiple linear regression analysis was used to test the correlation between the survival-associated pathways and TMB/NAL. Considering the consistency of TMB and NAL data, the pathways with a suitable β coefficient were retained as comutated (comut^+^) pathways.

Survival analysis (OS or PFS) for the pathway comutation status was performed by Kaplan–Meier curves, and their significance was assessed by the log-rank method. Fisher’s exact test was used to identify the association between the pathway comutation status and ORR.

### Differential analysis of immune-related features between pathway comut^+^ and comut^−^ patients

We first collected immune response-related genes, including immune checkpoints, T-cell receptor genes, tumour microenvironment (TME)-related genes, interferon-gamma (IFNγ)-associated genes, etc., from the published articles [39–41], which include 47 genes and were listed in Additional file 1: Table S3. For gene expression data of TCGA cancer patients, FPKM-normalized profiles were used, and all expression values were then log2(value + 1) transformed. We used the Wilcoxon rank-sum test to compare the differential expression levels of these immune response-related genes between comut^+^ and comut^−^ patients. The resulting p values were corrected using the FDR method.

Then, we compared the differences in immune-related signature features, including immune score, cytolytic activity (CYT) score, major histocompatibility complex (MHC)-I score, and T-cell-inflamed immune gene expression profile (GEP) score, between comut^+^ and comut^−^ patients. The immune score was estimated by the “ESTIMATE” R package [42]. The CYT score [43] and MHC-I score [44] were calculated by taking the mean expression of their signature genes. The GEP score [45] was estimated by performing ssGSEA using the GEP gene list.

Furthermore, the GSEA method was applied to identify the pathways associated with pathway comutation status. Specifically, a ranked gene list was produced based on the t-score of expression values between comut^+^ and comut^−^ patients, and then the functional gene sets or pathways were mapped to the ranked list. We performed these analyses using the “clusterProfiler” package [46].

### Meta-analysis of ORR and OS or PFS based on pathway comutation status across multiple immunotherapy cohorts

To pool outcomes across multiple immunotherapy cohorts, the random-effect inverse-variance weighted approach was employed, which is computed by hazard ratio (HR) and its 95% confidence interval (CI) to assess the magnitude of influence on therapeutic benefits such as ORR, PFS, and OS based on pathway comut^+^ status. We used I^2^ statistics to assess the heterogeneity across different immunotherapy cohorts. P_heterogeneity_ > 0.1 and I^2^ < 50% indicated that there was no significant inter-cohort heterogeneity, and the results were consistent in these cohorts.

## Results

### Identification of comutated signaling pathways to estimate TMB and NAL

**Fig. 1:**
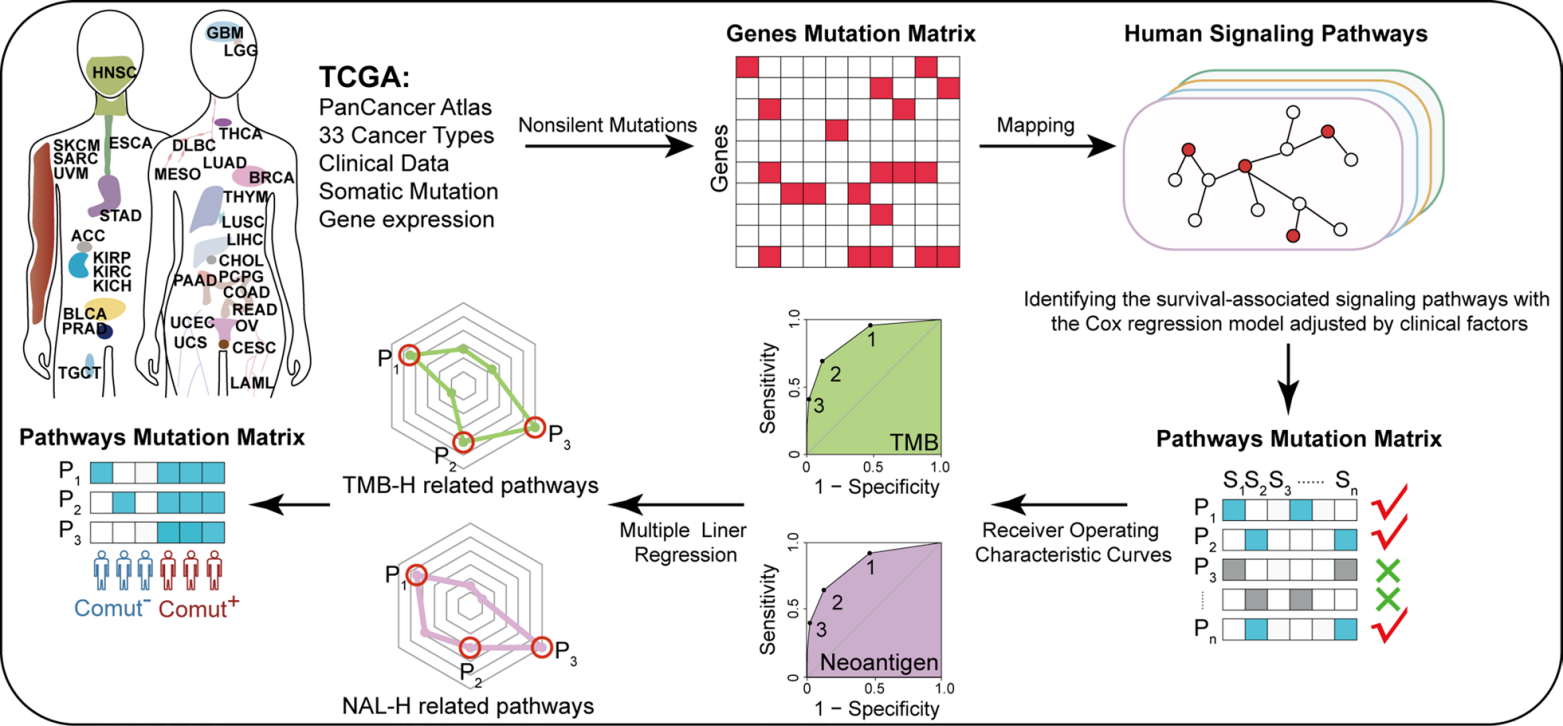
Flowchart to identify comutations in signaling pathways

A detailed flow chart of our study is shown in Fig. 1. In the TCGA cohort of 9763 patients across 33 cancer types, genes with a non-silent mutation in at least 1% of cancer patients were retained. Then, the genes were mapped to the 68 signaling pathways, and if any one of the genes involved in the pathway was mutated, the pathway was defined as mutated. To explore whether the mutations in signaling pathways could predict higher TMB and NAL, we grouped patients into two subgroups: “Pathway^+^”, those with mutations in one or more signaling pathways; “Pathway^−^”, those without mutations in any of the signaling pathways. Comparing the TMB and NAL levels between the two subgroups, we found that both TMB and NAL levels were significantly higher in the Pathway^+^ patient subgroup than in the Pathway^−^ patient subgroup by analysing the sequencing data from TCGA (Additional file 1: Fig. S1A, B), suggesting that tumours with more pathway mutations may generate and express more tumour-specific peptides, which are defined as neoantigens. Neoantigens are subsequently expressed on the surface of tumour cells via the major histocompatibility complex (MHC), which can activate a T-cell-mediated cytotoxic anti-tumour immune response and drive T-cell population expansion, thereby potentially affecting the immunotherapy response. These results indicate that comutations of multiple signaling pathways can be used to predict higher TMB and NAL levels.

**Fig. 2:**
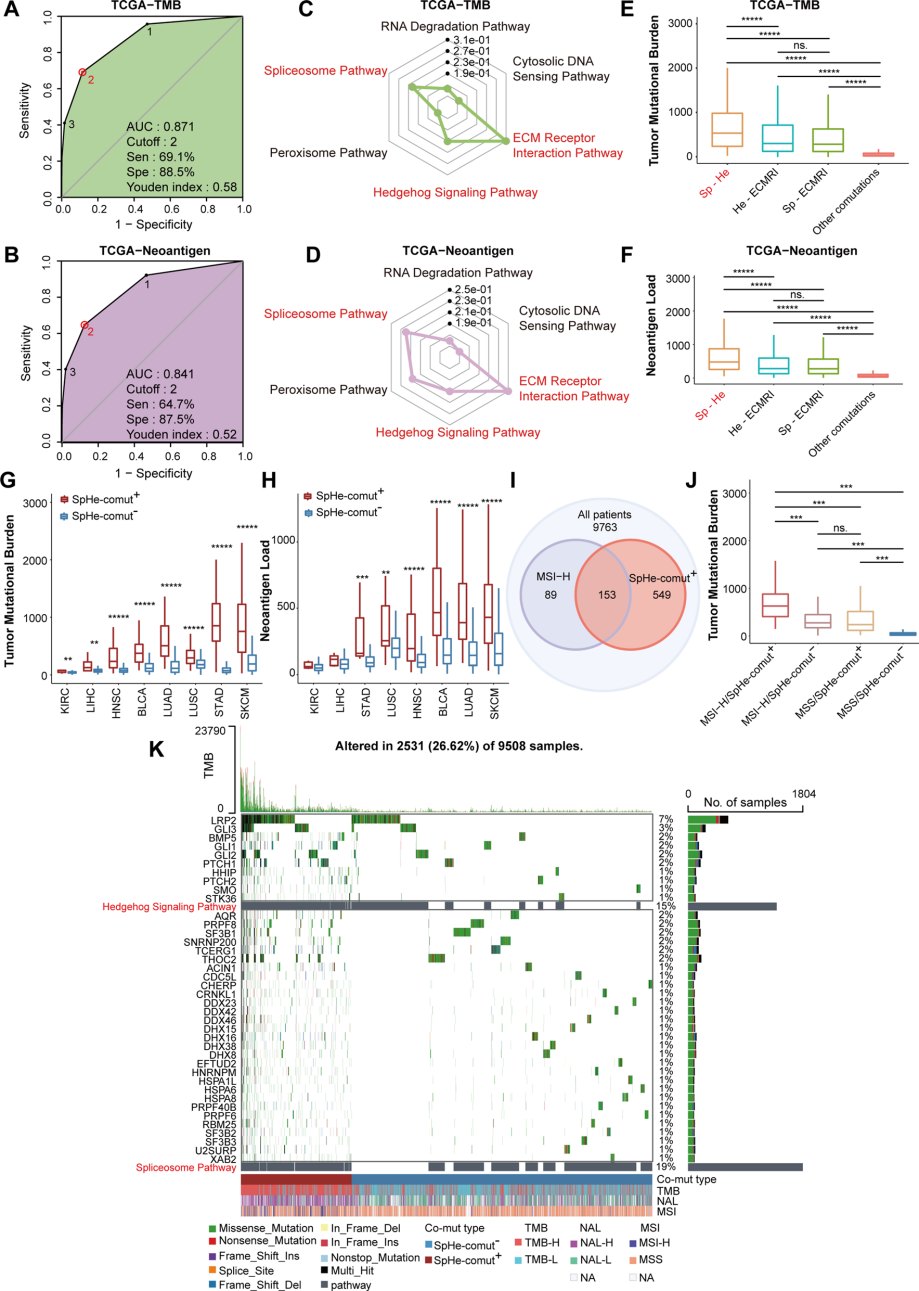
Correlations between SpHe-comut status and TMB, NAL, and MSI-H.** A**, **B** ROC curves of the number of comutated signaling pathways to predict higher TMB (**A**) and higher NAL levels (**B**) from TCGA. **C**, **D** Multiple linear regression β coefficients between six signaling pathways and TMB (**C**) and NAL (**D**). **E**, **F** Comparison of TMB (**E**) and NAL (**F**) between patients with Sp-ECMRI comutation, He-ECMRI comutation, Sp-He comutation, and other comutations. **G**, **H** Comparison of TMB (**G**) and NAL (**H**) between SpHe-comut^+^ and SpHe-comut^−^ groups in tumors with the approved indication of immune therapy. **I** Overlapping of patients with SpHe-comut^+^ and MSI-H. **J** Comparison of TMB in different combinations of SpHe-comut status (SpHe-comut^+^ and SpHe-comut^−^) and MSI status (MSI-H and MSS) groups. **K** Waterfall plot of two signature mutation pathways and the mutation genes involved in these pathways. *p < 0.05; **p < 0.01; ***p < 0.001; ****p < 0.0001; *****p < 0.00001. *BLCA* bladder urothelial carcinoma, *HNSC* head and neck squamous cell carcinoma, *KIRC* kidney renal clear cell carcinoma, *LIHC* liver hepatocellular carcinoma, *SKCM* skin cutaneous melanoma, *STAD* stomach adenocarcinoma, *LUAD* lung adenocarcinoma, *LUSC* lung squamous cell carcinoma

As not all signaling pathways are associated with cancer, we screened the survival-associated pathways. Specifically, for each pathway, we used the multivariate Cox proportional hazard model to estimate the association between the pathway mutation status and overall survival (OS) by accounting for known covariates, including patient age, sex, and cancer type. With FDR < 0.05, six significant pathways, such as the ECM receptor interaction (ECMRI) pathway, Spliceosome (Sp) pathway, Hedgehog (He) signaling pathway, and RNA degradation pathway, etc., were used for the following analysis (Additional file 1: Table S4). To further analyse how many pathways could be jointly used to predict higher TMB and NAL levels, receiver operating characteristic (ROC) curves were used to determine the optimal number of pathways in the pathway combination. To do this, we first classified the TCGA cancer patients into high TMB/NAL (TMB-H/NAL-H) and low TMB/NAL (TMB-L/NAL-L) groups according to the top quartile of TMB/NAL levels. When compared with other combinations of pathways in TMB-H estimation, mutations covering two pathways demonstrated the best Youden index, with 61.9% sensitivity and 88.5% specificity (Fig. 2A). Furthermore, the TCGA NAL data showed a similar result, with a sensitivity of 64.7% and specificity of 87.5% (Fig. 2B). These results indicated that the comutation of two signaling pathways may better predict higher TMB and NAL levels.

Considering latent interactions between different signaling pathways, we then used a multiple linear regression approach to determine the most efficient mode of pathway combination to research the relationships of signaling pathway mutations with TMB or NAL. As a result, the Spliceosome (Sp) pathway, Hedgehog (He) signaling pathway, and the ECMRI pathway, which have suitable β coefficients and contribute more to both TMB and NAL, were identified (Fig. 2C, D). To obtain the optimal two pathways, we compared the TMB and NAL for each pair of comutation pathway combinations. Despite not being able to completely exclude the effects of other comutation combinations, patients who had comutation of Sp-He illustrated significantly higher TMB and NAL than those with comutation of Sp-ECMRI and He-ECMRI or other comutations (Fig. 2E, F). Therefore, we defined the comutation in Sp-He pathways as “SpHe-comut^+^”; otherwise, we defined it as “SpHe-comut^−^“. By comparing the TMB and NAL levels between the “SpHe-comut^+^” and “SpHe-comut^−^” groups, we discovered that SpHe-comut^+^ patients showed significantly higher TMB and NAL than SpHe-comut^−^ patients (Additional file 1: Fig. S1C, D).

To explore the distribution of SpHe-comut status, we found that 702 patients were defined as SpHe-comut^+^ (7.19%) among the 9763 cases in the TCGA database. The number of SpHe-comut^+^ patients varied among different cancer types, ranging from 0% in KICH to 25.89% in SKCM (Additional file 1: Fig. S1E). We found that the distribution of TMB and NAL levels also varied among cancer types (Additional file 1: Fig. S1F, G), similar to the distribution of SpHe-comut^+^ among cancer types. We then analysed whether the presence of SpHe-comut^+^ was associated with an increased level of TMB or NAL. The results showed that there were significantly higher TMB (Fig. 2G) and NAL (Fig. 2H) levels in SpHe-comut^+^ patients than in SpHe-comut^−^ patients across cancer types with an approved indication of immune checkpoint inhibitors (such as SKCM, LUAD, HNSC, and BLCA, among others), except for the NAL levels of KIRC and LIHC. This may be due to the relatively low NAL levels in KIRC and LIHC (Fig. 2H), resulting in the difference not reaching statistical significance. Additionally, similar results were obtained for other cancer types (Additional file 1: Tables S5 and S6). Moreover, researchers found that MSI was strongly associated with TMB, and we then analysed whether the SpHe-comut^+^ status could be used to predict MSI-high (MSI-H) status. The results showed that SpHe-comut^+^ contributed significantly to MSI-H (Fisher’s exact test, P < 0.001) and predicted MSI-H with a sensitivity of 63.22% and a specificity of 94.15% (Fig. 2I). We then found that patients in the MSI-H/SpHe-comut^+^ group had significantly higher TMB than those in the MSI-H/SpHe-comut^−^ group, and in comparing microsatellite stability (MSS)/SpHe-comut^+^ to MSS/SpHe-comut^−^, a significant difference in TMB was also observed (Fig. 2J). Furthermore, the waterfall plot showed that the patients in the SpHe-comut^+^ group had higher TMB and NAL levels, which covered more cancer patients than the MSI-H group (Fig. 2K). These findings suggested that SpHe-comut^+^ could be used to substitute for TMB estimation and could predict more TMB-H patients than MSI-H patients.

### Correlations between SpHe-comut^+^ and immune-related features

**Fig. 3:**
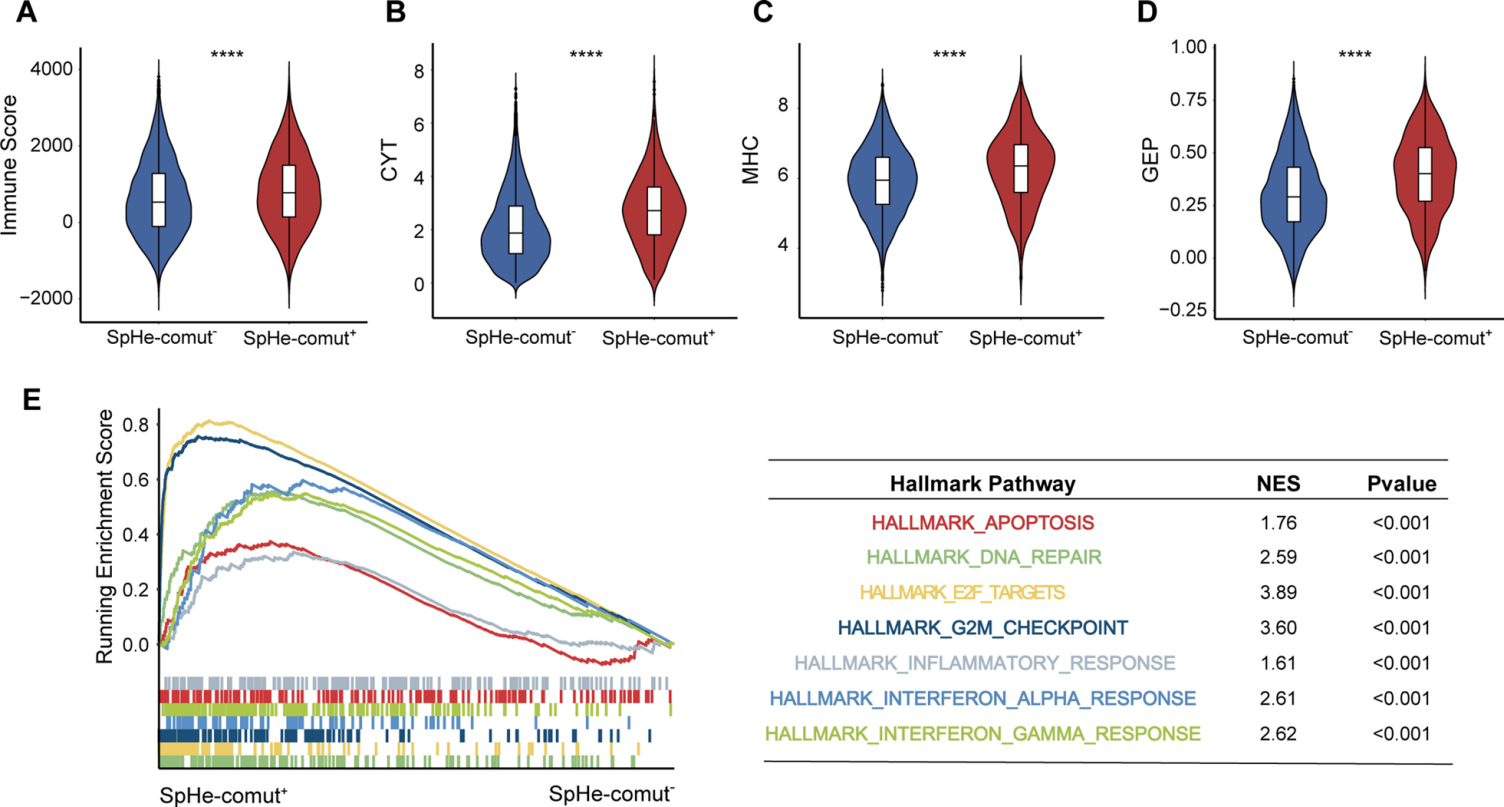
Correlation between SpHe-comut status and immune-related features.** A**–**D** Comparison of the immune score (**A**), CYT score (**B**), MHC-I score (**C**), and GEP score (**D**) between the SpHe-comut^+^ and SpHe-comut^−^ groups. **E** GSEA plot of significant hallmark pathways in comparison between the SpHe-comut^+^ and SpHe-comut^−^ groups. ****p < 0.0001

Previous studies have reported a connection between TMB and immune-related features, including immune score, CYT score, major histocompatibility complex (MHC)-I score, and T-cell-inflamed immune GEP score [43–45]. To explore whether the SpHe-comut statuses are also associated with those immune-related features, we analysed 9272 samples of 33 cancers from TCGA with both gene expression and somatic mutation data. The Immune Score was first calculated using the “ESTIMATE” algorithm, and our results showed that the patients with SpHe-comut^+^ had a significantly higher immune score than patients with SpHe-comut^−^ over a pan-cancer analysis (Fig. 3A). The CYT score was then calculated based on the mean transcript levels of granzyme A (GZMA) and perforin 1 (PRF1), which are significantly upregulated with cytotoxic T-cell activation [43]. The MHC-I score was calculated using the mean expression of MHC-I core genes, and the T-cell-inflamed immune GEP score was estimated by performing ssGSEA using the GEP gene list (Additional file 1: Table S4). We observed obviously higher CYT scores, MHC-I scores, and GEP scores in the SpHe-comut^+^ patient group than in the SpHe-comut^−^ patient group (Fig. 3B–D). It was reported that the production of MHC is a crucial component of the immune system, which presents invading pathogen peptides to the surface membrane of T cells and triggers the immune response [47]. The GEP score was reported as a biomarker to classify patients who can benefit from anti-PD-1 therapy [45]. These findings indicated that SpHe-comut^+^ was associated with immune-related features, suggesting that SpHe-comut^+^ may affect the efficacy of immunotherapy.

To further explore the biological functions affected by SpHe-comut^+^, GSEA was performed. A ranked gene list was constructed based on the gene differential expression levels (T-score) between the SpHe-comut^+^ and SpHe-comut^−^ groups across all cancer patients, and the gene sets of hallmark pathways from the MsigDB database were mapped to the ranked list. The results revealed that several immune-related hallmark pathways were significantly enriched in the SpHe-comut^+^ patient group, such as the interferon-gamma (IFNγ) response, DNA repair, and inflammatory response pathways (Fig. 3E). IFNγ was proposed to be an important marker of response to ICB in lung cancer and melanoma patients [48]. Deficiency in the DNA damage response has recently emerged as a major driver of tumour immunogenicity [49]. The inflammatory response (inflammation) has been demonstrated to be closely associated with all stages of development as well as with the efficacy of anti-cancer immunotherapy [50].

**Fig. 4:**
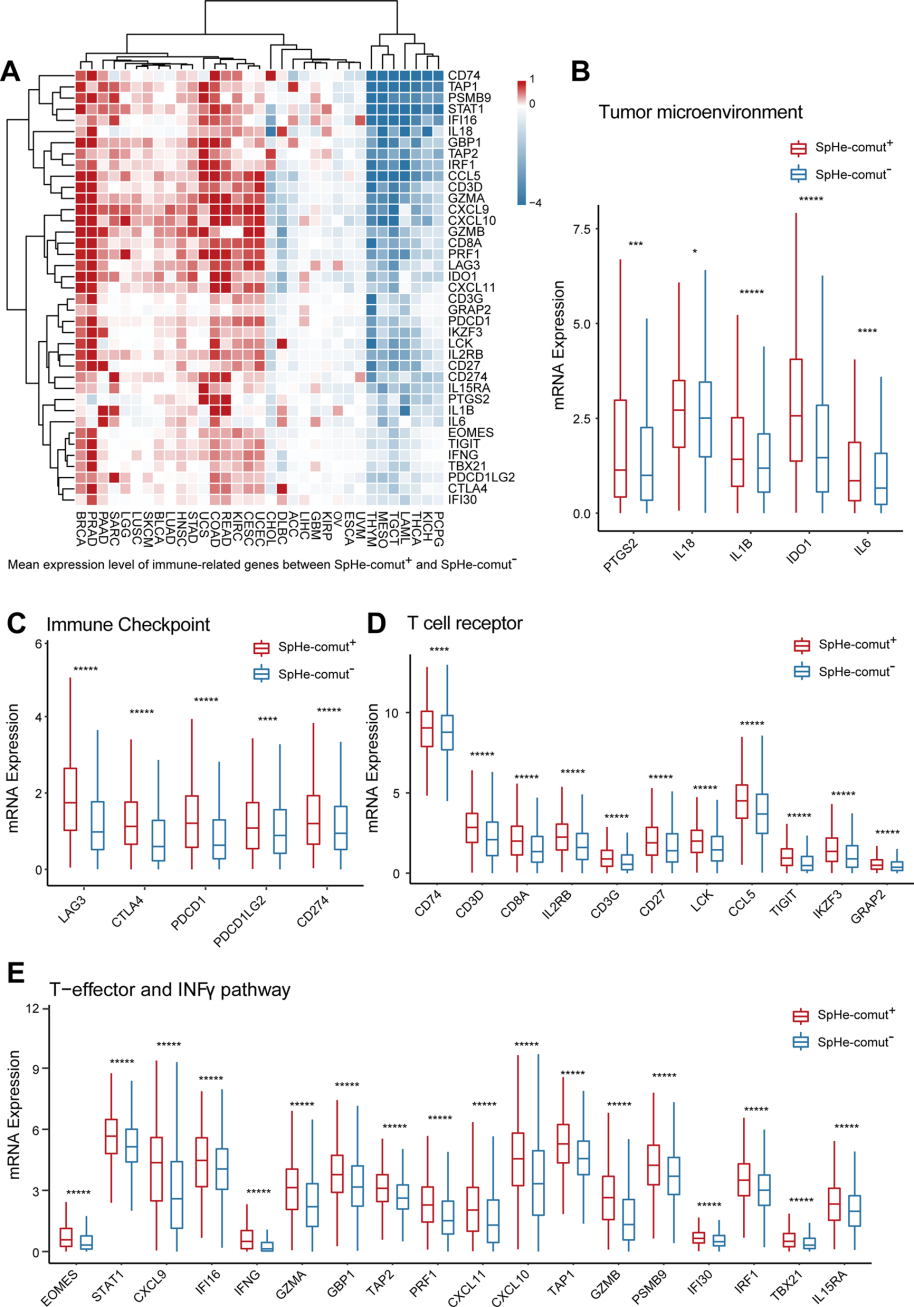
Correlations between SpHe-comut status and immune response-related gene mRNA expression.** A** Heatmap of mean expression differences in immune response-related genes between SpHe-comut^+^ and SpHe-comut^−^ patients across different cancer types. **B**–**E** Comparison of the expression levels of genes related to the tumour microenvironment (**B**), immune checkpoints (**C**), T-cell receptor (**D**), and INFγ pathway and T-effector (**E**) signature between SpHe-comut^+^ and SpHe-comut^−^ groups in the pan-cancer analysis. *FDR < 0.05; **FDR < 0.01; ***FDR < 0.001; ****FDR < 0.0001; *****FDR < 0.00001.

Additionally, we investigated the differential expression levels of immune response-related genes between the SpHe-comut^+^ and SpHe-comut^−^ groups over a pan-cancer analysis, and 39 of 47 genes showed significant differences (Wilcoxon rank-sum test, FDR < 0.05). We then compared the mean expression levels of these 39 genes between the SpHe-comut^+^ and SpHe-comut^−^ groups in each cancer type. Interestingly, the heatmap of mean difference expression levels of these genes showed that the cancer types with an approved indication of immune therapy (such as SKCM, LUAD, HNSC, and BLCA, among others) clustered together, and these genes presented higher mean expression levels in SpHe-comut^+^ patients in these cancer types (Fig. 4A). Moreover, in the pan-cancer analysis, these 39 genes, including five tumour microenvironment genes (Fig. 4B), five immune checkpoints (Fig. 4C), 11 T-cell receptors (Fig. 4D), and 18 T-effector and IFNγ pathway-relevant genes (Fig. 4E), were significantly up-regulated in the SpHe-comut^+^ group compared with the SpHe-comut^−^ group. These genes, such as IL18, IL1β, IL6, IL2RB, and IL15RA, are involved in the interleukin 1 (IL-1) family, which has been reported to promote the innate immune response to shape and improve the patient’s adaptive immune response [51]. These findings indicated that SpHe-comut^+^ patients may tend to respond to immunotherapy.

### SpHe-comut^+^ is a predictive and prognostic biomarker of ICBs

**Fig. 5:**
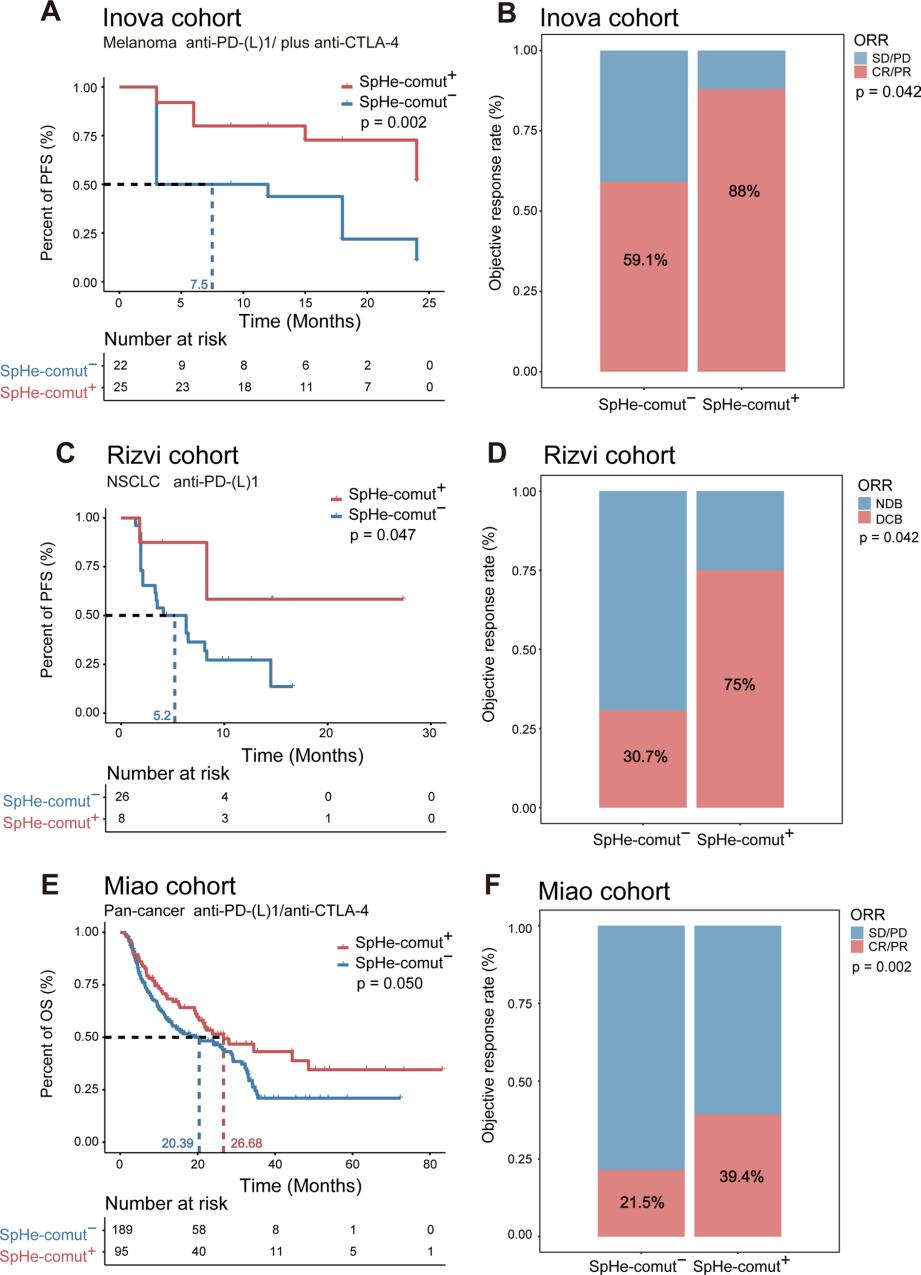
Comparisons of PFS (or OS) and ORR between SpHe-comut^+ ^and SpHe-comut^−^ groups across different cohorts.** A** Kaplan–Meier survival curves of PFS comparing SpHe-comut^+^ and SpHe-comut^−^ groups among patients with melanoma treated with anti-PD-(L)1 or anti-CTLA-4 therapy from the Inova cohort. **B** Comparison of ORR between the SpHe-comut^+^ and SpHe-comut^−^ groups from the Inova cohort. **C** Kaplan–Meier survival curves of PFS comparing the SpHe-comut^+^ and SpHe-comut^−^ groups in patients with NSCLC treated with anti-PD-1 therapy from the Rizvi cohort. **D** Comparison of ORR between the SpHe-comut^+^ and SpHe-comut^−^ groups from the Rizvi cohort. **E** Kaplan–Meier survival curves of OS comparing SpHe-comut^+^ and SpHe-comut^−^ groups in patients with pan-cancer treated with anti-CTLA4 or anti-PD-(L)1 therapy from the Miao cohorts. **F** Comparison of ORR between the SpHe-comut^+^ and SpHe-comut^−^ groups from the Miao cohort

To explore whether the SpHe-comut^+^ status can be used to predict the efficacy of immunotherapy, three immunotherapy cohorts were obtained from published studies for this analysis, including the Inova cohort (melanoma), the Rizvi cohort (NSCLC), and the Miao cohort (pan-cancer) (see “Materials and methods”). The SpHe comutation status was examined in these three independent immunotherapy cohorts, and then the patients were divided into SpHe-comut^+^ and SpHe-comut^−^ groups. Our results showed that patients with SpHe-comut^+^ status had significantly higher TMB and NAL levels in these three cohorts, which was consistent with the results previously observed in TCGA pan-cancer analyses (Additional file 1: Fig. S2A–C). Moreover, we also found that the SpHe-comut^+^ status can be used as a biomarker to predict the efficacy of immunotherapy in three cohorts. Specifically, in the Inova cohort with 50 melanoma patients treated with anti-PD-(L)1 plus anti-CTLA4, 25 patients were defined as SpHe-comut^+^, and those patients had a significantly longer PFS (median PFS, SpHe-comut^+^ not reached (NR) vs. SpHe-comut^−^ 7.5 months; HR, 0.27; 95% CI, 0.11–0.64; log-rank test p = 0.002; Fig. 5A). An obviously higher ORR was found in the SpHe-comut^+^ patient group (SpHe-comut^+^ 88.0% vs. SpHe-comut^−^ 59.1%; Fisher’s exact test, p = 0.042; Fig. 5B). In the NSCLC Rizvi cohort, a significant association between SpHe-comut^+^ and longer PFS was observed (median PFS, SpHe-comut^+^ not reached (NR) vs. SpHe-comut^−^ 5.2 months; HR, 0.31; 95% CI 0.091–1.1; log-rank p = 0.047; Fig. 5C), as well as higher ORR (SpHe-comut^+^ 75% vs. SpHe-comut^−^ 30.7%; Fisher’s exact test, p = 0.042; Fig. 5D). The same results were observed in the Miao cohort, in which a longer OS (median OS, SpHe-comut^+^ 26.68 vs. SpHe-comut^−^ 20.39 months; HR, 0.69; 95% CI 0.47–1; log-rank p = 0.050; Fig. 5E) and a higher ORR (SpHe-comut^+^ 39.4% vs. SpHe-comut^−^ 21.5%; Fisher’s exact test, p = 0.002; Fig. 5F) was found in the SpHe-comut^+^ patient group. These results suggested that SpHe-comut^+^ had a good prognostic predictive ability in three immunotherapy cohorts and can be used as a predictor for the efficacy of immunotherapy.

To further verify the prognostic power of SpHe-comut^+^, the other four public immunotherapy cohorts with sufficient information on genomic alterations and survival were analysed. The four public immunotherapy cohorts included (1) the Hellmann cohort of 75 NSCLC patients treated with anti-PD-1 plus anti-CTLA4; (2) the Allen and Snyder cohort, a pooled analysis of two cohorts of 174 metastatic melanoma patients treated with anti-CTLA4; and (3) the MSKCC cohort of 1661 pan-cancer patients treated with anti-PD-(L)1 or anti-CTLA4. Our analysis revealed a significant positive association between SpHe-comut^+^ and TMB and NAL levels (Additional file 1: Fig. S2D–F).

**Fig. 6:**
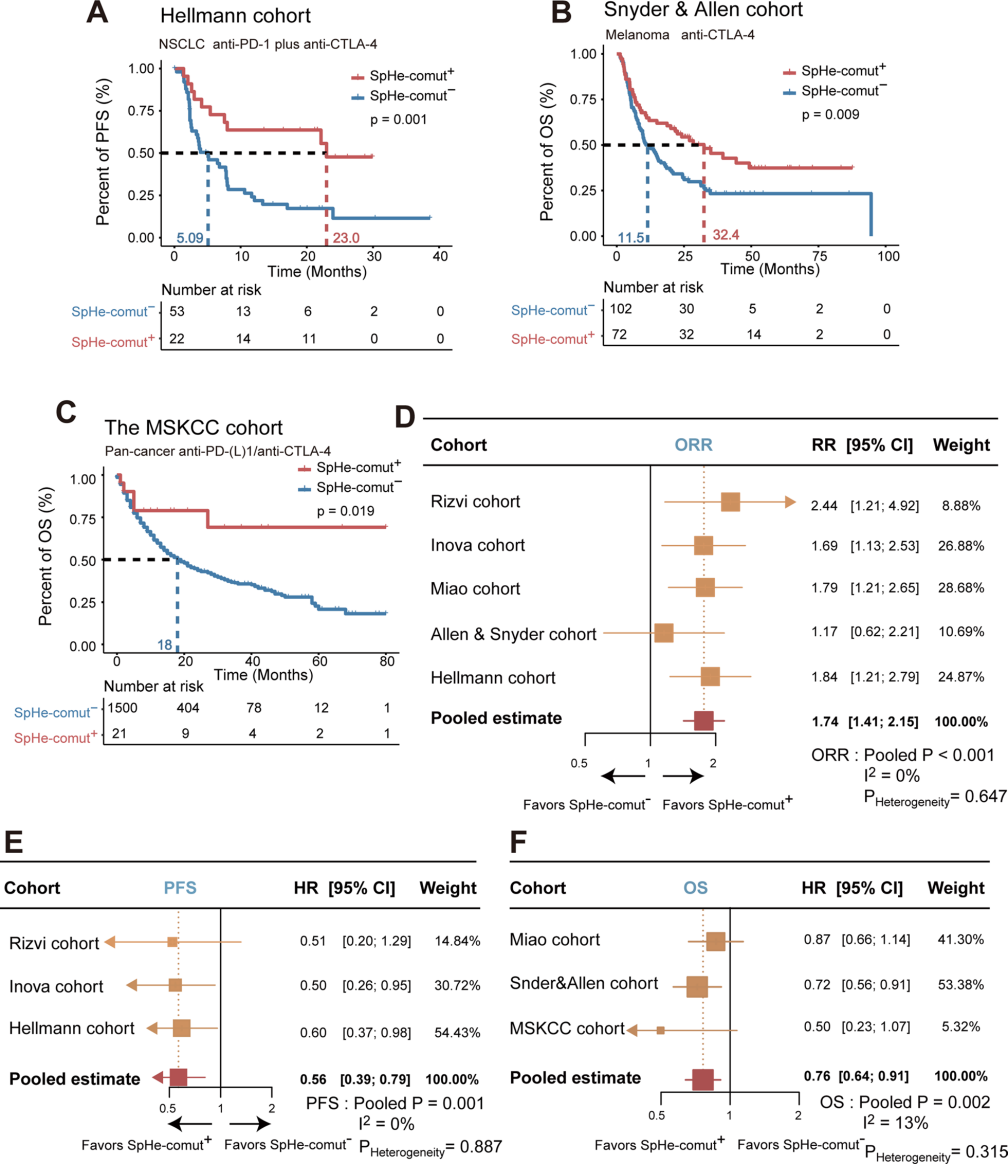
Pooled analysis of PFS, OS, and ORR comparing the SpHe-comut^+^ and SpHe-comut^−^ patients across different cohorts.** A** Kaplan–Meier survival curves of PFS comparing the SpHe-comut^+^ and SpHe-comut^−^ groups in the Hellmann cohort. **B**, **C** Kaplan–Meier survival curves of OS comparing the SpHe-comut^+^ and SpHe-comut^−^ groups in the Allen and Snyder cohort (**B**) and the MSKCC cohort (**C**). **D**–**F** Pooled estimates of ORR (**D**), PFS (**E**), and OS (**F**) comparing SpHe-comut^+^ and SpHe-comut^−^ patients across different cohorts

Next, a survival analysis was performed in the Hellmann, Allen and Snyder, and MSKCC immunotherapy cohorts to explore the impacts of SpHe-comut status on PFS and OS. The survival analysis showed that SpHe-comut^+^ was significantly associated with a better prognosis in these immunotherapy cohorts (Fig. 6A–C). The SpHe-comut^+^ patients showed a higher ORR than the SpHe-comut^−^ patients in the Hellmann and Allen and Snyder cohorts (Additional file 1: Fig. S3A, B). According to a pooled analysis for the above cohorts, compared to the SpHe-comut^−^ group, the SpHe-comut^+^ patient group showed a higher ORR [pooled risk ratio (RR), 1.74; 95% CI 1.41–2.15; p < 0.001, Fig. 6D], a longer PFS (pooled HR, 0.56; 95% CI 0.39–0.79; p = 0.001, Fig. 6E), and a longer OS (pooled HR, 0.76; 95% CI 0.64–0.91; p = 0.002, Fig. 6F). The results of the statistical analysis for heterogeneity in all pooled estimates were insignificant (P_heterogeneity_ > 0.10 and I^2^ < 50%), indicating the consistency of the association between SpHe-comut^+^ status and favourable benefits to ICBs across these cohorts.

Furthermore, we performed multivariate Cox regression analyses of SpHe-comut status and survival by adjusting for clinical features (such as patient age, sex, etc.) in the Inova cohort, Rizvi cohort, and Miao cohort. In each cohort, we found that SpHe-comut was an independent prognostic factor (Additional file 1: Fig. S4A–C).

### Comparison of SpHe-comut with other immunotherapy biomarkers

Previous studies highlighted the relevance of TMB to the response to ICB therapy across a wide variety of cancer types [35]. Recently, Wang et al. found that comutations in DDR pathways can also serve as potential biomarkers for ICB [20]. We used the concordance index (C-index) and ROC curve analysis to evaluate the predictive value of SpHe-comut status for response to ICB therapy and compared it with TMB and DDR-comut status. In the Inova cohort (melanoma), the C-index and ROC curve analyses demonstrated that SpHe-comut status was a predictive biomarker of immunotherapy clinical benefit (C-index = 0.69, AUROC = 0.69), and its predictive power was superior to that of TMB and DDR-comut status (Additional file 1: Fig. S5A, B). Similar results were observed in the Rizvi cohort (NSCLC) and Miao cohort (pan-cancer) (Additional file 1: Fig. S5A, B).

**Fig. 7:**
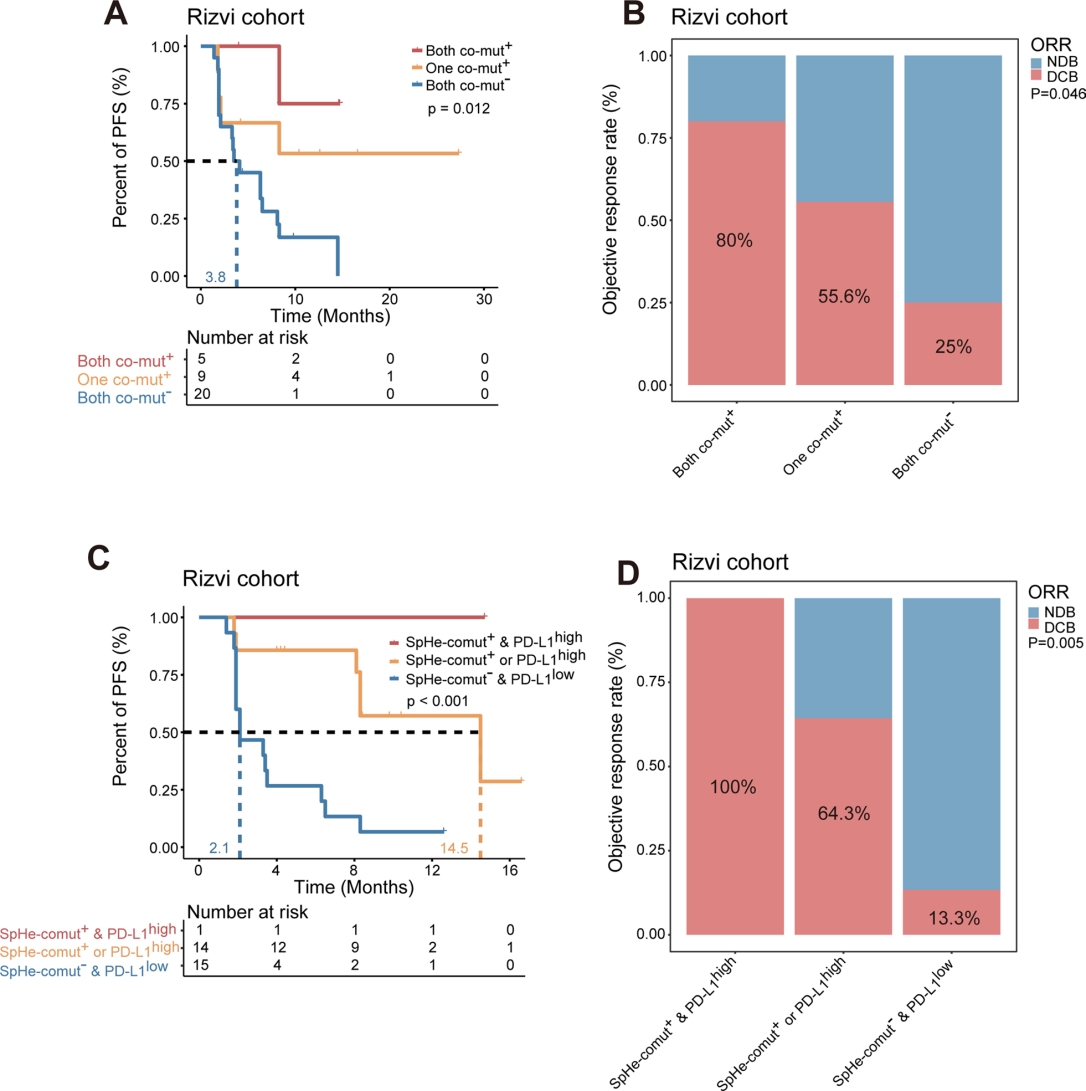
Combining SpHe-comut status with DDR-comut status and PD-L1 expression for the prediction of ICB therapy.** A** Kaplan–Meier survival curves of PFS comparing the patients within each of the three indicated subgroups classified by SpHe-comut status and DDR-comut status in the Rizvi cohort. **B** Comparison of proportion of patients with DCB calculated within each of the three indicated subgroups classified by SpHe-comut status and DDR-comut status in the Rizvi cohort. **C** Kaplan–Meier survival curves of PFS comparing the patients within each of the three indicated subgroups classified by SpHe-comut status and PD-L1 expression in the Rizvi cohort. **D** Comparison of proportion of patients with DCB calculated within each of the three indicated subgroups classified by SpHe-comut status and PD-L1 expression in the Rizvi cohort

Considering that SpHe-comut and DDR-comut are both immunotherapy predictive biomarkers based on TMB-screened pathway mutations, we then explored the joint utility of these two biomarkers for patient stratification. Survival analysis in the Rizvi cohort showed that dual-positive SpHe-comut and DDR-comut (both comut^+^) patients had longer PFS than either single-positive (single comut^+^) or dual-negative (both comut^−^) patients (median PFS, not reached (NR) vs. not reached (NR) vs. 3.8 months, log-rank test, p = 0.012, Fig. 7A). Additionally, there was an increased proportion of DCB in both comut^+^ groups compared to the other two groups (80% vs. 55.6% vs. 25%, p = 0.046, Fig. 7B). We obtained similar results for the Inova cohort (Additional file 1: Fig. S6A, B) and Miao cohort (Additional file 1: Fig. S6C, D), which heralded the possibility of integrating SpHe-comut and DDR-comut as a novel marker for predicting the immune response.

A number of recent studies have illustrated the importance of PD-L1 expression in response to PD-(L)1 blockade immunotherapy [52]. In the Rizvi cohort, we collected data across the board from patients with valid PD-L1 expression information. According to the cut-off point of 50%, the patients were classified into PD-L1^high^ and PD-L1^low^ groups. Based on multivariable Cox analysis, SpHe-comut status (HR, 0.16; 95% CI 0.04–0.67; p = 0.012) and PD-L1 expression (HR, 0.091; 95% CI 0.021–0.39; p = 0.001) were two independent predictor variables for anti-PD-(L)1 treatment (Additional file 1: Fig. S4B). We thus examined the relationship between these two biomarkers. A Kaplan–Meier analysis of the Rizvi cohort revealed that patients with dual positive status for SpHe-comut and PD-L1 (SpHe-comut^+^ and PD-L1^high^) obtained more favourable PFS than either single positive (SpHe-comut^+^ or PD-L1^high^) or dual negative patients (SpHe-comut^−^ and PD-L1^low^, median PFS, not reached (NR) vs. 14.5 vs. 2.1 months, log-rank test, p < 0.001, Fig. 7C). In addition, the objective response analysis showed an increased proportion of DCB in the SpHe-comut^+^ and PD-L1^high^ groups compared to the two other groups (100% vs. 64.3% vs. 13.3%, p = 0.005, Fig. 7D). Based on these results, we speculated that the addition of SpHe-comut to PD-L1 might further improve the predictive value for anti-PD-(L)1 therapy.

In addition, we collected some mutated markers that have been regarded as stable immunotherapy markers in NSCLC, including those of the Li et al. method [18] and Bai et al. method [53]. Li et al. suggested that highly mutated NOTCH signaling may serve as a biomarker for the prediction of the prognosis of NSCLC patients treated with ICIs, and Bai et al. developed an eight-gene GMS risk model to predict the immunotherapeutic benefit in non-squamous NSCLC. We applied the Li et al. method and Bai et al. method in the Rizvi cohort, and the patients were then divided into GMS-high and GMS-low, NOTCH-high and NOTCH-low groups. We found that there was no significant difference in patients’ progression-free survival and immunotherapy response between each of the two patient groups (GMS-high vs. GMS-low or NOTCH-high vs. NOTCH-low, Additional file 1: Fig. S7A–D), while our method could classify patients into two groups with significant distinctive progression-free survival and immunotherapy response (Fig. 5C, D). These observations indicate that SpHe-comut had a better prognostic predictive ability than the Li et al. and Bai et al. methods.

## Discussion

Recently, immunotherapy with immune checkpoint blockades (ICBs) has emerged as a promising treatment for cancer. TMB is a biomarker that can be used to predict patient response to cancer immunotherapy, indicating that patients with high TMB have better responses to ICB therapy. An increased neoantigen load is associated with a better response to ICB treatment as well. However, these indicators have limited use due to the lack of a validated cut-off value. Cancer is a disease in which cells acquire the ability to divide and proliferate uncontrollably, usually through genetic changes in specific genes. Mutation profiles differ dramatically between individuals with cancer. They may be used as indicators for treatment response and disease progression. Applying a molecular pathway-based approach to cancer mutations can dramatically boost their biomarker potential. Therefore, investigation of genomic mutation pathways may lead to the discovery of effective biomarkers for immunotherapy.

Alterations in human signaling pathways have been shown to be related to the development of cancer and its immunotherapy by many studies [21]. As we used the clinical immunotherapy data of cancer patients to identify the comutations in signaling pathways, the results may be easier to translate into clinical applications. In the study, we demonstrated that the presence of comutations in the Spliceosome (Sp) pathway and Hedgehog (He) signaling pathway could be used as a novel biomarker for ICB therapy. The prognostic and predictive values of SpHe-comut^+^ were validated in seven independent immunotherapy cohorts. In the melanoma cohort, the SpHe-comut^+^ group exhibited longer median PFS (Fig. 5A) and better ORR (Fig. 5B). In the NSCLC cohort, both the median PFS (Fig. 5C) and ORR (Fig. 5D) were improved in the SpHe-comut^+^ group. Moreover, in the pan-cancer cohort, the median OS and ORR were improved in the SpHe-comut^+^ group (Fig. 5E, F). Meanwhile, the predictive power of the response to immunotherapy of SpHe-comut^+^ was demonstrated to be superior to those of DDR-comut^+^ and TMB (Additional file 1: Fig. S5A, B). To further verify the prognostic power of SpHe-comut^+^, four other public immunotherapy cohorts were adopted. According to a pooled analysis for the above cohorts, the results showed that the statistical analysis for heterogeneity in the ORR, OS, and PFS pooled estimates were insignificant (P_heterogeneity_ > 0.10 and I^2^ < 50%), indicating the consistency of the association between SpHe-comut^+^ status and clinical results of ICBs across these cohorts (Fig. 6D–F).

As there are still multiple cellular and molecular mechanisms involved in immunotherapy, we thus combined SpHe-comut^+^ with other biomarkers, such as DDR-comut^+^ and PD-L1 expression. The results showed that patients with both comut^+^ statuses (SpHe-comut^+^ and DDR-comut^+^) had longer survival and higher response proportions to immunotherapy than either single-positive (single comut^+^) or dual-negative (both comut^−^) patients in the Rizvi, Inova, and Miao cohorts, respectively (Fig. 7A, B, Additional file 1: Fig. S6A–D). Moreover, in the Rizvi cohort with PD-L1 expression information, we combined SpHe-comut and PD-L1 expression and demonstrated that patients with dual-positive status (SpHe-comut^+^ and PD-L1^high^) or single-positive status (SpHe-comut^+^ or PD-L1^high^) obtained more favourable PFS and DCB than patients with dual-negative status (SpHe-comut^−^ and PD-L1^low^ status) (Fig. 7C, D). Based on these results, we speculated that the combination of SpHe-comut and DDR-comut or PD-L1 may operate as a synergistic component in immunotherapy prediction.

The advantage of our method is that it provides an optional and cost-effective approach by offering a small panel of genes in the signature pathways that can easily be translated into an easy-to-use clinical assay to identify potential immunotherapy responders. However, there are still limitations in our research. For example, we did not differentiate with respect to whether the gene mutations in the pathway were functional. Otto Warburg suggested that defects in mitochondrial respiration may be the underlying cause of cancer [54], and it would be better to add the role of comutation of mitochondrial genes to the prediction of the clinical outcomes of immunotherapy in our study. Our attempt to recruit functional mutations into our comut^+^ pattern was handicapped by the limited information available regarding the functions of different mutations. This suggests that the development of immunotherapeutic biomarkers requires more advanced sequencing techniques and nanomaterials, such as carbon nanotubes [55, 56]. If there is more information available regarding the functions of different mutations, the comut^+^ pattern may provide higher accuracy for efficacy prediction for ICB delivery.

## Conclusion

In summary, the pan-cancer analysis revealed that comutation of Spliceosome (Sp) pathways and the Hedgehog (He) signaling pathway (defined as SpHe-comut^+^) was associated with increased TMB and NAL levels, as well as increased levels of immune-related signatures. Furthermore, the patients with SpHe-comut^+^ exhibited a higher ORR and a longer OS or PFS than SpHe-comut^−^ patients in seven independent immunotherapy cohorts. Our results suggest that SpHe-comut^+^ might provide an optional and cost-effective approach for identifying potential immunotherapy responders, which might complement recent immunotherapy biomarkers.

## Supplementary Information

**Additional file 1: Figure S1.** Correlations between co-mutations of signaling pathways and tumor mutational burden, neoantigen load. **Figure S2.** Correlations between SpHe-comut status and Tumor mutational burden or neoantigen load. **Figure S3.** Comparison of the objective response rate between the SpHe-comut^+^ and SpHe-comut^−^ groups. **Figure S4.** Univariable and multivariable Cox analysis of SpHe-comut status and clinicopathological factors (Age, Sex, and PD-L1 expression) for PFS or OS in the ICBs cohorts. **Figure S5.** Comparison of performance for predicting ORR among SpHe-comut, DDR-comut and TMB as a predictive biomarker. **Figure S6.** Combining SpHe-comut status with DDR-comut status for the prediction of ICB therapy. **Figure S7.** Comparison of SpHe-comut status with NOTCH and GMS for the prediction of ICB therapy in the Rizvi cohort. **Table S1.** Data source. **Table S3.** Gene list of immune-related gene signature. **Table S4.** The significant survival-associated signaling pathways identified by the multivariate Cox regression model adjusted by clinical factors. **Table S5.** Comparison of tumor mutational burden between SpHe-comut^+^ and SpHe-comut^−^ groups across different cancer types. **Table S6.** Comparison of neoantigen load between SpHe-comut^+^ and SpHe-comut^−^ groups across different cancer types.

**Additional file 2: Table S2.** The Information for time period and dose selection of involved cohorts.

## Data Availability

The TCGA cancer patients’ data is downloaded from the GDC TCGA data portal (https://portal.gdc.cancer.gov/). The seven immunotherapy cohorts are available in the publications cited in the manuscript.

## Abbreviations

ACC: Adrenocortical carcinoma
BLCA: Bladder urothelial carcinoma
BRCA: Breast invasive carcinoma
CESC: Cervical squamous cell carcinoma and endocervical adenocarcinoma
CHOL: Cholangiocarcinoma
COAD: Colon adenocarcinoma
CTLA4: Cytotoxic T-lymphocyte antigen-4
C-index: Concordance index
CI: Confidence interval
CR: Complete response
CYT: Cytolytic activity
DLBC: Lymphoid neoplasm diffuse large B-cell lymphoma
DCB: Durable clinical benefit
DDR: DNA damage response
ESCA: Esophageal carcinoma
ECMRI pathway: ECM receptor interaction pathway
GEP: Gene expression profile
FPKM: Fragments per kilobase of exon model per million mapped fragments
FDR: False discovery rate
GBM: Glioblastoma multiforme
HNSC: Head and neck squamous cell carcinoma
IFNγ: Interferon-gamma
HR: Hazard ratio
ICB: Immune checkpoint blockades
IL-1: Interleukin 1
KICH: Kidney chromophobe
KIRC: Kidney renal clear cell carcinoma
KIRP: Kidney renal papillary cell carcinoma
KEGG: Kyoto encyclopedia of genes and genomes
LAML: Acute myeloid leukemia
LGG: Brain lower grade glioma
LIHC: Liver hepatocellular carcinoma
LUAD: Lung adenocarcinoma
LUSC: Lung squamous cell carcinoma
MESO: Mesothelioma
MSI: Microsatellite instability
MSS: Microsatellite stability
MHC: Major histocompatibility complex
NDB: No durable benefit
NAL: Neoantigen load
NSCLC: Non-small cell lung cancer
OV: Ovarian serous cystadenocarcinoma
OS: Overall survival
ORR: Objective response rate
PD: Progressive disease
PR: Partial response
PAAD: Pancreatic adenocarcinoma
PCPG: Pheochromocytoma and paraganglioma
PRAD: Prostate adenocarcinoma
PD-1: Programmed cell death 1
PFS: Progression-free survival
READ: Rectum adenocarcinoma
ROC: Receiver operating characteristic
SARC: Sarcoma
SKCM: Skin cutaneous melanoma
STAD: Stomach adenocarcinoma
STES: Stomach and esophageal carcinoma
SD: Stable disease
ssGSEA: Single-sample GSEA
TMB: Tumor mutational burden
GSEA: Gene set enrichment analysis
TGCT: Testicular germ cell tumors
THCA: Thyroid carcinoma
THYM: Thymoma
TCIA: The cancer immunome atlas
TCGA: The cancer genome atlas
TME: Tumor microenvironment
UCEC: Uterine corpus endometrial carcinoma
UCS: Uterine carcinosarcoma
UVM: Uveal melanoma
WES: Whole-exome sequencing
