# Supplementary material for: Identification of comutation in signaling pathways to predict the clinical outcomes of immunotherapy
Source: J Transl Med. 2022 Dec 23;20:613. doi: 10.1186/s12967-022-03836-3 (PMC9783967; doi:10.1186/s12967-022-03836-3)
Supplement: Supplementary file 1 — Additional file 1: Figure S1. Correlations between co-mutations of signaling pathways and tumor mutational burden, neoantigen load. Figure S2. Correlations between SpHe-comut status and Tumor mutational burden or neoantigen load. Figure S3. Comparison of the objective response rate between the SpHe-comut+ and SpHe-comut− groups. Figure S4. Univariable and multivariable Cox analysis of SpHe-comut status and clinicopathological factors (Age, Sex, and PD-L1 expression) for PFS or OS in the ICBs cohorts. Figure S5. Comparison of performance for predicting ORR among SpHe-comut, DDR-comut and TMB as a predictive biomarker. Figure S6. Combining SpHe-comut status with DDR-comut status for the prediction of ICB therapy. Figure S7. Comparison of SpHe-comut status with NOTCH and GMS for the prediction of ICB therapy in the Rizvi cohort. Table S1. Data source. Table S3. Gene list of immune-related gene signature. Table S4. The significant survival-associated signaling pathways identified by the multivariate Cox regression model adjusted by clinical factors. Table S5. Comparison of tumor mutational burden between SpHe-comut+ and SpHe-comut− groups across different cancer types. Table S6. Comparison of neoantigen load between SpHe-comut+ and SpHe-comut− groups across different cancer types. [file 12967_2022_3836_MOESM1_ESM.pdf]

Additional file 1

Identification of comutation in signaling pathways to predict the clinical outcomes of immunotherapy

Contents:

Figure S1-S7

Table S1-S6

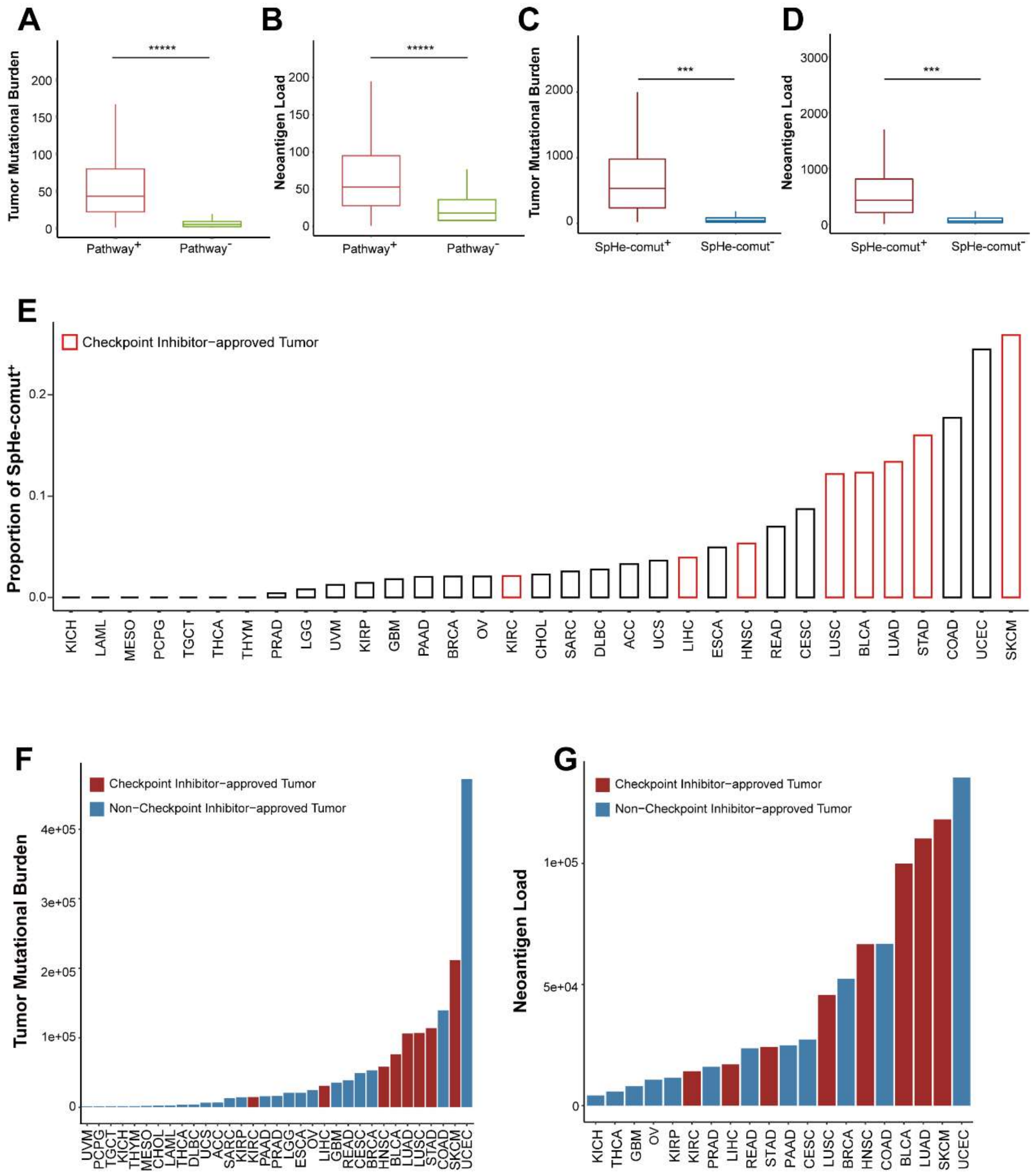

Figure S1. Correlations between co-mutations of signaling pathways and tumor mutational burden, neoantigen load.

(A) Comparison of tumor mutational burden between mutated and wild-type signaling pathways subgroups. **(B)** Comparison of neoantigen load between mutated and wild-type signaling pathways subgroups. **(C)** Comparison of tumor mutational burden between SpHe-comut^{+} and SpHe-comut^{-} subgroups. **(D)** Comparison of neoantigen load between SpHe-comut^{+} and SpHe-comut^{-} subgroups. **(E)** Frequency of SpHe-comut^{+} in different TCGA cancer types. **(F)** Frequency of tumor mutational burden in different TCGA cancer types. **(G)** Frequency of neoantigen load in different TCGA cancer types. *, $p < 0.05$; **, $p < 0.01$; ***, $p < 0.001$; ****, $p < 0.0001$; *****, $p < 0.00001$.

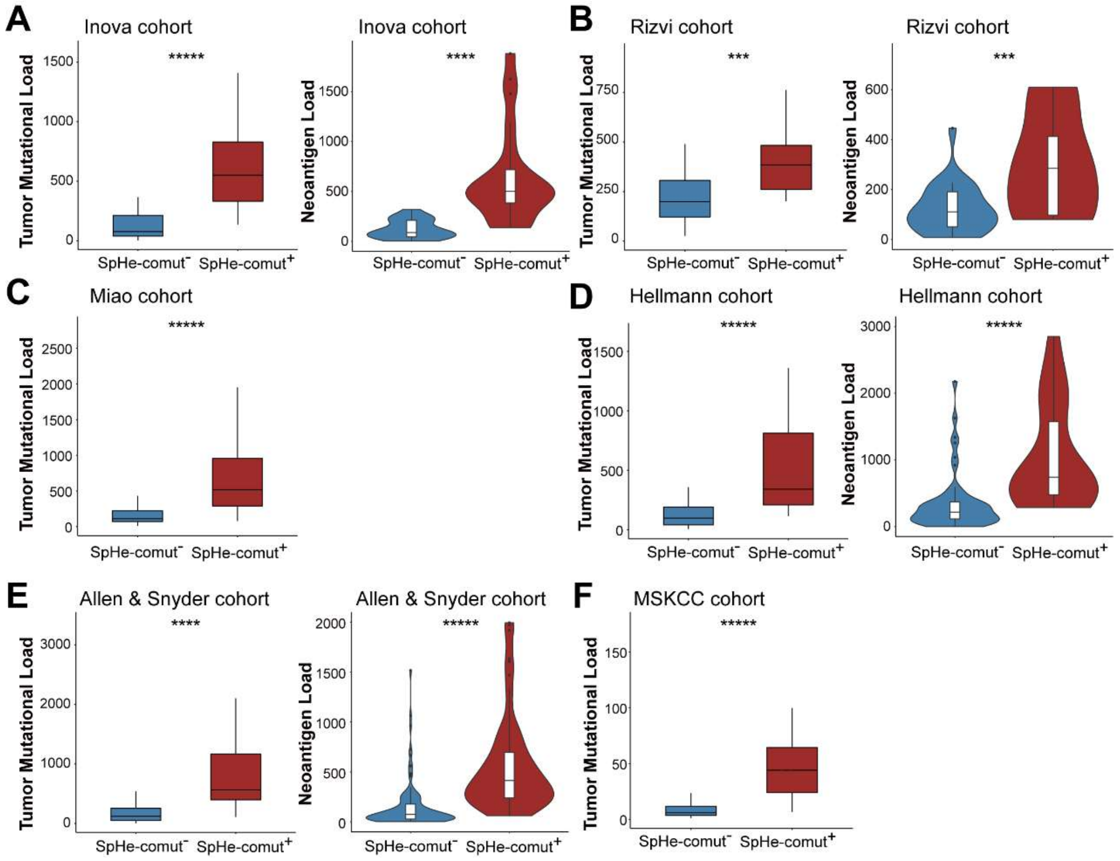

Figure S2. Correlations between SpHe-comut status and Tumor mutational burden or neoantigen load.

(A)-(F) Comparisons of tumor mutational burden and neoantigen load in SpHe-comut^{+} and SpHe-comut^{-} subgroups from the Inova cohort (**A**), the Rizvi cohort (**B**), the Miao cohort (**C**), the Hellmann cohort (**D**), the Allen & Snyder cohort (**E**), and the MSKCC cohort (**F**). ***, $p < 0.001$; ****, $p < 0.0001$; *****, $p < 0.00001$.

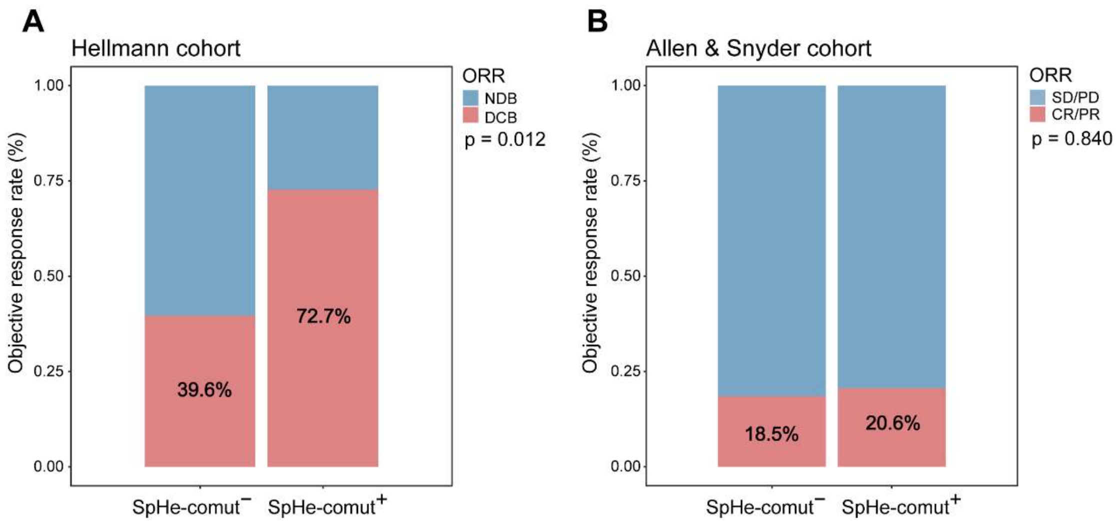

Figure S3. Comparison of the objective response rate between the SpHe-comut$^+$ and SpHe-comut$^-$ groups.

(A) Comparison of proportion of patients with DCB between the SpHe-comut$^+$ and SpHe-comut$^-$ subgroups from the Hellmann cohort. (B) Comparison of the objective response rate between the SpHe-comut$^+$ and SpHe-comut$^-$ groups in the Allen & Snyder cohort.

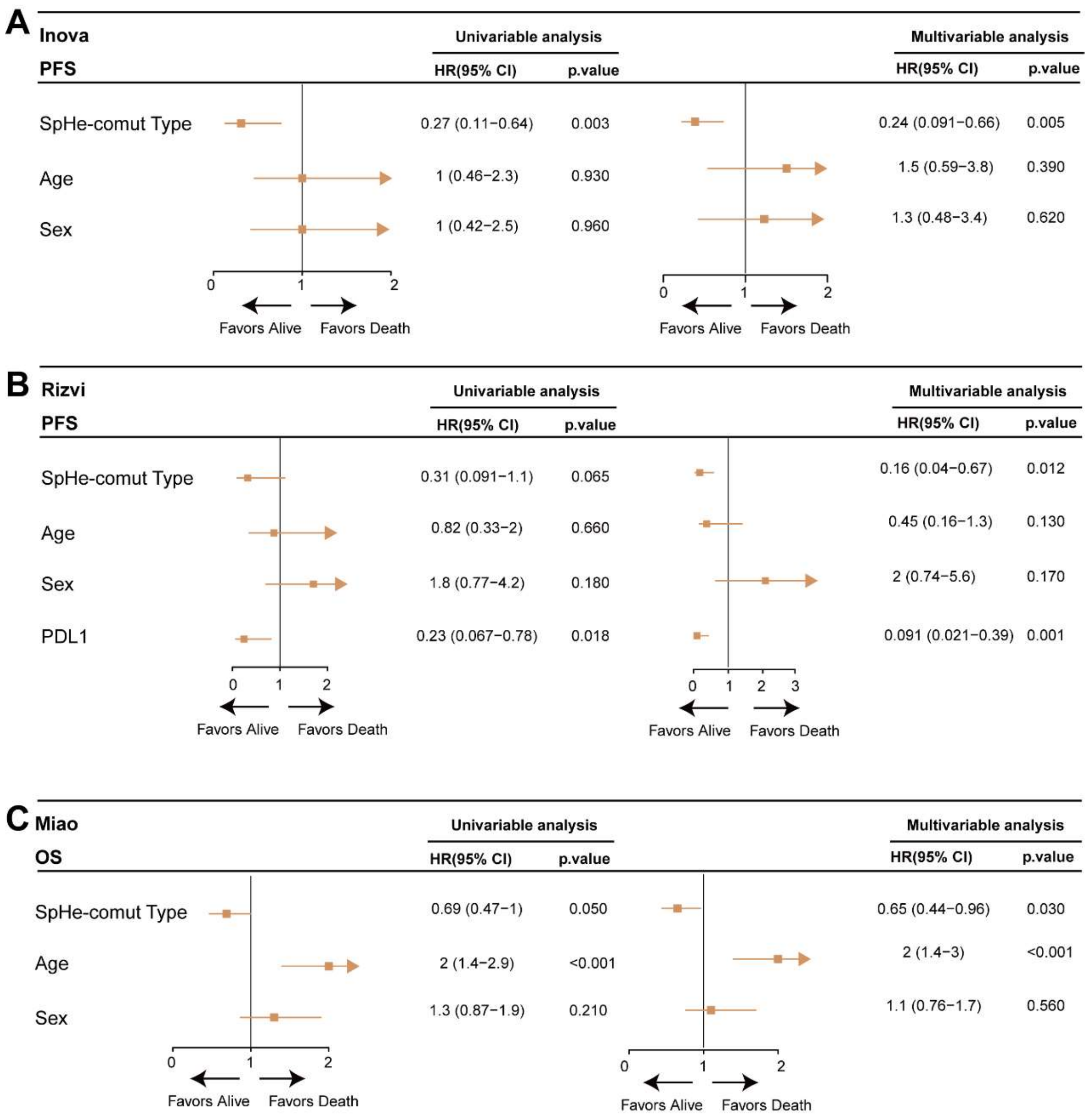

Figure S4. Univariable and multivariable Cox analysis of SpHe-comut status and clinicopathological factors (Age, Sex, and PD-L1 expression) for PFS or OS in the ICBs cohorts.

(A) Univariable and multivariable Cox analyses of SpHe-comut status and clinicopathological factors (Age, Sex) for PFS in the Inova cohort. **(B)** Univariable and multivariable Cox analyses of SpHe-comut status and clinicopathological factors (Age, Sex, PD-L1 expression) for PFS in the Rizvi cohort. **(C)** Univariable and multivariable Cox analyses of SpHe-comut status and clinicopathological factors (Age, Sex) for OS in the Miao cohort.

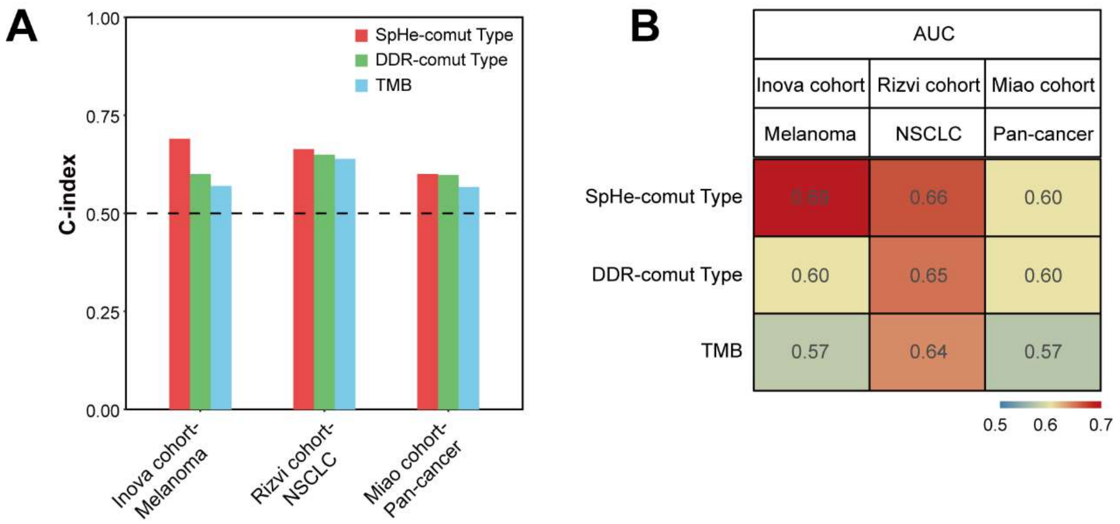

Figure S5. Comparison of performance for predicting ORR among SpHe-comut, DDR-comut and TMB as a predictive biomarker.

(A) Comparison of C-index for predicting ORR among SpHe-comut, DDR-comut and TMB in the Inova cohort (Melanoma), the Rizvi cohort (NSCLC), and the Miao cohort (Pan-cancer). **(B)** Comparison of AUC for predicting ORR among SpHe-comut, DDR-comut and TMB in the Inova cohort (Melanoma), the Rizvi cohort (NSCLC) and the Miao cohort (Pan-cancer).

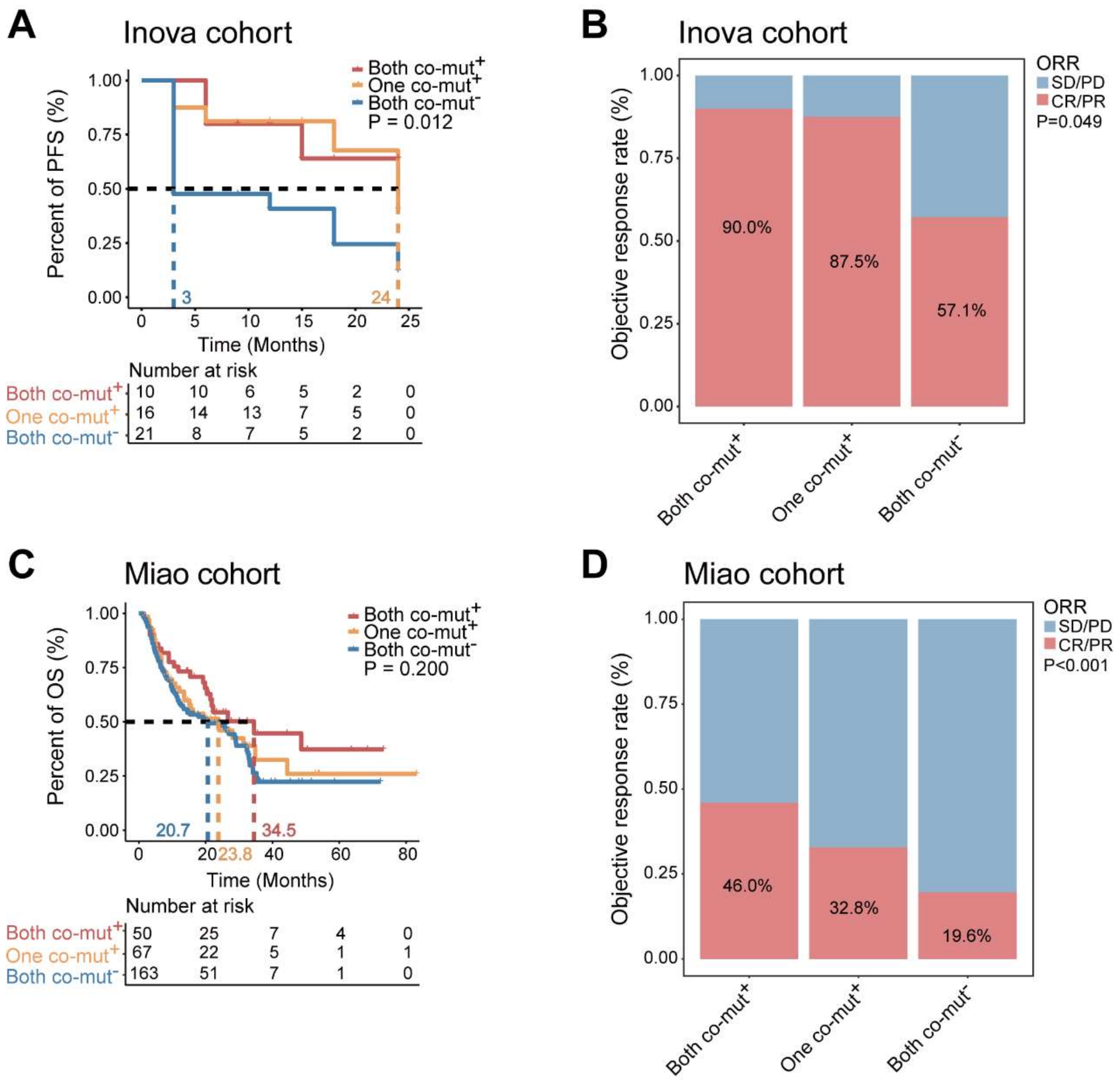

Figure S6. Combining SpHe-comut status with DDR-comut status for the prediction of ICB therapy.

(A) Kaplan-Meier estimates of PFS classified by the three indicated subgroups classified by SpHe-comut status and DDR-comut status in the Inova cohort. **(B)** Proportional representation of objective response rate among subgroups categorized by the three indicated subgroups classified by SpHe-comut status and DDR-comut status in the Inova cohort. **(C)** Kaplan-Meier estimates of OS classified by the three indicated subgroups classified by SpHe-comut status and DDR-comut status in the Miao cohort. **(D)** Proportional representation of objective response rate among subgroups categorized by the three indicated subgroups classified by SpHe-comut status and DDR-comut status in the Miao cohort.

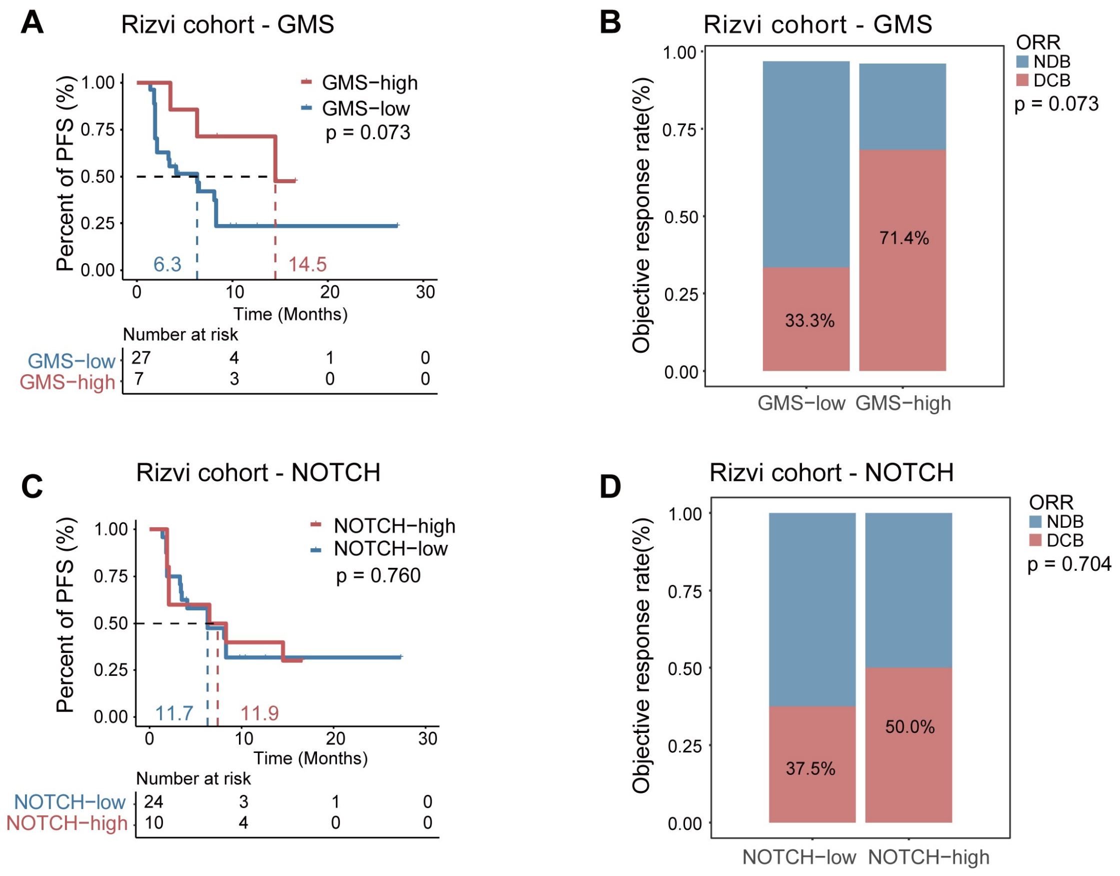

Figure S7. Comparison of SpHe-comut status with NOTCH and GMS for the prediction of ICB therapy in the Rizvi cohort.

A) Kaplan-Meier survival curves of PFS comparing the GMS-high and GMS-low groups. **(B)** Comparison of ORR between the GMS-high and GMS-low groups. **(C)** Kaplan-Meier survival curves of PFS comparing the NOTCH-high and NOTCH-low groups. **(D)** Comparison of ORR between the NOTCH-high and NOTCH-low groups.

Table S1. Data source.

Data source	Tumor	N	WES	RNAseq	Neoantigen	Clinical outcome
TCGA	33 cohorts	9763	9763	9272	5446	OS
Rizvi cohort	Non-small cell lung cancer with anti-PD-1 therapy	34	34	—	34	DCB PFS
Hellmann cohort	Non-small cell lung cancer with anti-PD-1 plus anti-CTLA-4 therapy	75	75	—	75	DCB PFS
Inova cohort	Melanoma with anti-PD-(L)1/ plus anti-CTLA-4 therapy	50	50	—	50	ORR PFS
Allen cohort	Melanoma with anti-CTLA-4 therapy	110	110	—	110	ORR OS
Snyder cohort	Melanoma with anti-CTLA-4 therapy	64	64	—	64	ORR OS
Miao cohort	Pan-cancer with anti-PD-1/ anti-PD-L1/ anti-CTLA-4 therapy	284	284	—	—	ORR OS
MSKCC cohort	Pan-cancer with anti-PD-1/ anti-PD-L1/ anti-CTLA-4 therapy	1661	1661	—	—	OS

TCGA, The Cancer Genome Atlas; WES, whole-exome sequencing; ORR, objective response rate; DCB, durable clinical benefit; PFS, progression-free survival; OS, Overall survival.

Table S2. The Information for time period and dose selection of involved cohorts.

(Full forms see the Additional file 2: Table S2.xlsx)

Table S3. Gene list of immune-related gene signature.

Classification	Genes
Immune checkpoint	PD-1, PD-L1, PD-L2, LAG3, CTLA4, TIM3, VTCN1
T-effector and INFγ pathway	GBP1, IFI16, IFI30, IFNG, IRF1, STAT1, TAP1, TAP2, FAS, PSMB9, IL15RA, GZMA, GZMB, EOMES, CXCL10, CXCL9, CXCL11, TBX21, PRF1
T cell receptor	CD27, GRAP2, LCK, PTPRCAP, CCL5, IL2RB, IKZF3, CD3G, CD74, CD3D, CD8A, CD4, TIGIT
Tumor microenvironment	IDO1, PTGS2, IL1B, IL18, IL6, IL12A, TNF, CD73
Cytolytic activity (CYT)	GZMA, PRF1
Major Histocompatibility complex (MHC)	HLA-A, HLA-B, HLA-C, TAP1, TAP2, NLRC5, PSMB9, PSMB8, B2M
Gene expression profile (GEP)	CCL5, CD27, CD274, CD276, CD8A, CMKLR1, CXCL9, CXCR6, HLA-DQA1, HLA-DRB1, HLA-E, IDO1, LAG3, NKG7, PDCD1LG2, PSMB10, STAT1, TIGIT

Table S4. The significant survival-associated signaling pathways identified by the multivariate Cox regression model adjusted by clinical factors.

Pathway	No. of Genes	HR (95% CI for HR)	P value	FDR
Spliceosome pathway	127	0.82 (0.75 - 0.9)	4.2e-05	0.001
Hedgehog signaling pathway	56	0.85 (0.77 - 0.94)	1.5e-03	0.029
ECM receptor interaction pathway	84	0.88 (0.81-0.95)	1.7e-03	0.029
RNA degradation pathway	59	0.83 (0.73 - 0.94)	3.9e-03	0.044
Peroxisome pathway	78	0.73 (0.64 - 0.83)	1.1e-06	< 0.001
Cytosolic DNA sensing pathway	55	0.78 (0.66 - 0.92)	2.8e-03	0.038

Table S5. Comparison of tumor mutational burden between SpHe-comut$^+$ and SpHe-comut$^-$ groups across different cancer types.

Tumor	SpHe-comut$^-$			SpHe-comut$^+$			Pvalue
	N	mean	SD	N	mean	SD	
ACC	88	1.08	1.62	3	28.22	17.2	0.004
BLCA	356	3.88	3.39	50	12.48	12.69	<0.001
BRCA	941	1.16	1.58	20	15.34	25.89	<0.001
CESC	251	2.73	3.30	24	25.11	54.83	<0.001
CHOL	43	1.00	1.67	1	15.76	-	0.098
COAD	311	3.07	4.44	67	40.46	36.79	<0.001
DLBC	35	2.78	1.85	1	1.87	-	0.630
ESCA	173	2.57	1.32	9	11.11	15.25	0.002
GBM	373	1.16	0.60	7	70.95	115.04	0.001
HNSC	479	2.56	2.18	27	11.50	14.76	<0.001
KICH	65	0.61	1.85	-	-	-	-
KIRC	325	1.15	0.88	7	1.60	0.65	0.048
KIRP	272	1.37	0.66	4	1.61	0.75	0.524
LAML	114	0.62	2.03	-	-	-	-
LGG	494	0.59	0.31	4	62.24	115.76	0.001
LIHC	341	2.09	1.47	14	7.20	9.75	0.003
LUAD	421	4.28	4.11	65	15.23	8.84	<0.001
LUSC	418	5.35	3.85	58	9.89	9.31	<0.001
MESO	78	0.67	0.67	-	-	-	-
OV	423	1.46	0.90	9	3.79	5.78	0.041
PAAD	145	0.70	0.43	3	105.61	180.18	0.006
PCPG	175	0.18	0.12	-	-	-	-
PRAD	473	0.55	0.63	2	83.75	94.62	0.015
READ	120	2.27	1.08	9	83.12	101.62	<0.001
SARC	228	1.05	1.30	6	16.24	14.87	<0.001
SKCM	335	6.52	6.48	117	28.90	37.87	<0.001
STAD	336	3.29	4.71	64	29.57	27.01	<0.001
TGCT	127	0.31	0.20	-	-	-	-

THCA	482	0.19	0.15	-	-	-	-
THYM	107	0.43	1.65	-	-	-	-
UCEC	395	3.44	5.20	128	86.39	107.45	<0.001
UCS	53	1.23	0.57	2	53.91	50.33	0.019
UVM	79	0.26	0.10	1	8.11	-	0.090

Table S6. Comparison of neoantigen load between SpHe-comut^{+} and SpHe-comut^{-} groups across different cancer types.

Tumor	SpHe-comut^{-}			SpHe-comut^{+}			Pvalue
	N	mean	SD	N	mean	SD	
BLCA	340	206.17	188.59	46	643.80	530.74	<0.001
BRCA	832	52.20	105.91	17	526.35	816.06	<0.001
CESC	155	111.52	188.61	15	688.13	771.02	<0.001
COAD	167	130.04	412.10	43	1047.12	917.22	<0.001
GBM	144	55.79	29.90	-	-	-	-
HNSC	454	120.86	105.84	26	451.65	712.96	<0.001
KICH	65	64.74	124.31	-	-	-	-
KIRC	253	54.81	30.97	5	70.20	26.90	0.188
KIRP	157	73.34	45.38	-	-	-	-
LIHC	171	90.09	64.33	9	177.89	220.87	0.056
LUAD	399	186.66	173.68	59	605.93	474.61	<0.001
LUSC	151	238.61	195.39	21	460.71	435.89	0.002
OV	198	52.38	33.58	5	62.80	26.37	0.303
PAAD	119	80.26	64.94	3	5095	8608.33	0.017
PRAD	402	36.98	45.44	1	1152	-	0.089
READ	80	68.15	41.59	6	3031.33	3049.25	0.006
SKCM	247	231.29	252.77	84	725.92	715.22	<0.001
STAD	73	118.97	114.27	7	2225.14	3564.30	<0.001
THCA	372	15.75	15.55	-	-	-	-
UCEC	193	107.45	158.81	49	2339.94	3137.69	<0.001
